# XRN1-Resistant Elements Are Located in the 3′-Terminal Regions of Certain Genome Segments of Jingmenviruses

**DOI:** 10.3390/v18090946

**Published:** 2026-08-29

**Authors:** Egor V. Okhezin, Alexander G. Litov, Ivan S. Kholodilov, Galina G. Karganova

**Affiliations:** 1 Laboratory of Biology of Arboviruses, FSASI “Chumakov Federal Scientific Center for Research and Development of Immune-and-Biological Products of RAS” (Institute of Poliomyelitis), 108819 Moscow, Russia; oe-74@mail.ru (E.V.O.); ivan-kholodilov@bk.ru (I.S.K.); karganova@bk.ru (G.G.K.); 2 Department of Virology, Faculty of Biology, Lomonosov Moscow State University, 119234 Moscow, Russia; 3 Department of Organization and Technology of Production of Immunobiological Preparations, Institute for Translational Medicine and Biotechnology, Sechenov First Moscow State Medical University (Sechenov University), 119048 Moscow, Russia

**Keywords:** Alongshan virus, Yanggou tick virus, subgenomic RNA, XRN1

## Abstract

Jingmenviruses have been identified over the past decade. During this period, significant advancements have been made in understanding their diversity, biology, and genomic functionality. Conventionally characterized orthoflaviviruses are distinguished by a specific class of subgenomic RNAs (sfRNAs) that contain exoribonuclease-resistant RNA (xrRNA) structures. These sfRNAs are the products of incomplete 5′–3′ degradation of genomic RNA by the host exoribonuclease XRN1. Resistance to complete hydrolysis is mediated by highly conserved secondary structural motifs localized within the 3′ untranslated region (UTR) of the genomic RNA. In classical orthoflaviviruses, xrRNAs play key roles in regulating viral replication, modulating the host immune response, and driving host cell adaptation. In the present study, we evaluated the exonuclease resistance of the 3′ UTR genomic RNA segments of Alongshan and Yanggou viruses using in vitro assays. Our results demonstrate that 1 and 4 segments of Alongshan virus and 3 and 4 segments of Yanggou tick virus exhibit resistance to XRN1-mediated degradation.

## 1. Introduction

The genus *Orthoflavivirus* belongs to the family *Flaviviridae*, which encompasses several mosquito- and tick-borne human pathogens. Medically significant members include dengue virus (DENV) [1], yellow fever virus (YFV) [2], Japanese encephalitis virus (JEV) [3], Zika virus (ZIKV) [4], West Nile virus (WNV) [5], and tick-borne encephalitis virus (TBEV) [6].

Members of the genus Orthoflavivirus produce unique, small (300–500 nt) subgenomic non-coding RNAs (sfRNAs) derived from the 3′ untranslated region (3′ UTR) of the genomic RNA [7]. During infection, the viral genomic RNA undergoes 5′–3′ degradation by the host cytoplasmic exoribonuclease XRN1 [8,9]. However, complete degradation is halted by highly structured RNA motifs within the 3′ UTR known as exoribonuclease-resistant RNAs (xrRNAs) [7,8]. These elements form unique tertiary folds—often characterized by mechanical ring-like pseudoknots—that physically stall XRN1 progression without impairing viral RNA synthesis [8].

The resulting accumulation of sfRNAs plays a key role in viral pathogenesis and host immune evasion [9,10]. In vertebrate hosts, sfRNAs act as molecular decoys that suppress the type I interferon (IFN) response, for instance, by cooperating with viral proteins such as NS5 to inhibit STAT1 phosphorylation [11] or by sequestering host RNA-binding proteins [10]. In arthropod vectors, sfRNAs modulate host cellular defense mechanisms by counteracting RNA interference (RNAi) and suppressing apoptosis, thereby enhancing viral replication, persistence, and vector-borne transmission [9,10].

The recently identified genus *Jingmenvirus* (*Flaviviridae*) includes Jingmen tick virus (JMTV), Alongshan virus (ALSV), Yanggou tick virus (YGTV), Takachi virus, and Newport tick virus [12]. The Jingmenvirus genome comprises four positive-sense, single-stranded RNA (+ssRNA) segments that are capped and polyadenylated. Two of these segments encode non-structural proteins homologous to the NS3 and NS5 proteins of classical orthoflaviviruses, whereas the remaining segments encode structural proteins unique to this viral group. The JMTV (flavi-like Kindia tick virus) possesses 5′- and 3′-downstream AUG region (DAR) motifs, as well as a highly conserved 5′-CACAG-3′ pentanucleotide [13]. DAR motifs engage in base-pairing to circularize the viral genome, a necessary step for initiating viral replication. Additionally, conserved tetraloop structures within the 5′ and 3′ UTRs of Alongshan and Yanggou tick viruses have been reported [14].Alongshan virus has also been associated with human disease [15]. 

In this study, we demonstrate the in vitro generation of subgenomic RNAs from Alongshan and Yanggou tick viruses. Our results indicate that two of the four genomic segments of both ALSV and YGTV exhibit resistance to XRN1-mediated degradation.

## 2. Materials and Methods

### 2.1. Virus Cultivation in Cell Lines

Alongshan virus strain Miass-519, Yanggou tick virus strain Erzhin14-T20074 [16], and yellow fever virus vaccine strain 17D were obtained from our institute’s collection in the form of culture fluid from infected cells and used without additional passaging. The YFV strain 17D served as a positive control.

### 2.2. RNA Extraction

Total RNA was extracted from the culture supernatant of infected cells using 750 μL of TRI Reagent^®^ LS (Sigma-Aldrich, St. Louis, MO, USA) according to the manufacturer’s protocols. RNA was dissolved in 3 µL of water.

### 2.3. cDNA Synthesis and Amplification

Rapid amplification of cDNA ends (RACE) was performed using the Mint RACE cDNA Amplification Kit (Evrogen, Moscow, Russia) in accordance with the manufacturer’s protocol. The first-strand synthesis reaction mixture contained 3 μL of isolated RNA, 1 μL of the PlugOligo adapter (Evrogen, Moscow, Russia), 1 μL of 3′ primer (Evrogen, Moscow, Russia), 2 μL of 5× first-strand synthesis buffer, 1 μL of 20 mM DTT, 1 μL of 10 mM dNTPs, and 1 μL of Mint reverse transcriptase (Evrogen, Russia). The mixture was incubated at 42 °C for 30 min. Subsequently, IP solution was added, and incubation was continued at 42 °C for an additional 1.5 h. Amplification was performed using Encyclo DNA polymerase (Evrogen, Moscow, Russia) following the guidelines of the Mint RACE cDNA Amplification Kit. PCR was carried out using segment-specific oligonucleotides (Table 1) at a final concentration of 5 pmol/μL.

### 2.4. Molecular Cloning

The resulting PCR products were cloned into the pAL2-T TA-cloning vector (Evrogen, Moscow, Russia) at an insert-to-vector molar ratio of 3:1. Ligation was performed in a 10 μL reaction volume using T4 DNA ligase (SibEnzyme, Novosibirsk, Russia). The reaction mixture contained 1 μL of 10× ligation buffer, 2 μL of vector (50 ng), 1 μL of T4 ligase, and the target insert, and was brought to volume with ddH_2_O. The mixture was incubated at 4 °C for 16 h. Chemically competent *Escherichia coli* TOP10 cells were then transformed with the ligation mixture without further purification and plated onto 1.2% LB agar containing the appropriate selective antibiotics. White colonies selected via blue–white screening were inoculated into 3 mL of SOB medium supplemented with ampicillin (1 μg/mL). Cultures were grown overnight (12–16 h) at 37 °C with shaking at 180 rpm. Following incubation, the cell suspension was harvested by centrifugation at 8000 rpm for 5 min. The supernatant was discarded, and plasmid DNA was isolated using the GeneJET Plasmid Miniprep Kit (Thermo Scientific, Waltham, MA, USA).

### 2.5. Sanger Sequencing

Recombinant plasmids were sequenced using the M13(-21) universal primer (Applied Biosystems, Foster City, CA, USA) and custom M13_long_F primer. Sequencing reactions were performed using the ABI PRISM BigDye™ Terminator v. 3.1 Cycle Sequencing Kit on an ABI PRISM 3500 Genetic Analyzer (Applied Biosystems, Foster City, CA, USA). Sequence assembly and consensus analysis were conducted using SeqMan v.7.0.0 software (DNASTAR, Madison, WI, USA).

### 2.6. In Vitro Transcription

To generate RNA templates for transcription, oligonucleotides targeting the 3′ ends of all four segments of Alongshan virus, Yanggou tick virus, and yellow fever virus were employed (Table 1). For in vitro transcription of ALSV and YFV, the template consisted of a PCR product that had been cloned into the pAL2-T plasmid vector and subsequently excised using EcoRI (SibEnzyme, Novosibirsk, Russia) and Sfr303I (SibEnzyme, Novosibirsk, Russia) restriction endonucleases. In the case of YGTV, the template was a PCR product amplified from pAL2-T with gene-specific primers (Table 1). In vitro transcription was performed using the T7 High Efficiency Transcription Kit (TransGen Biotech, Beijing, China). The 20 μL reaction mixture included 1.6 μL of each NTP (100 mM stock), 7.1 μL of template DNA, and 0.5 μL of RNasin^®^ Ribonuclease Inhibitor (Promega, Madison, WI, USA). The mixture was incubated at 37 °C for 2 h. Following incubation, 2 μL of DNase I was added and incubated for 30 min at 37 °C to remove the template DNA; the reaction was terminated by adding 1 μL of 0.5 M EDTA. Synthetic RNA samples were further purified using TRI Reagent^®^ LS (Sigma-Aldrich, St. Louis, MO, USA) and stored at −80 °C. For Alongshan virus, independent RNA synthesis was performed for each replicate prior to enzymatic treatment. For Yanggou tick virus, independent in vitro enzymatic processing was performed using a single RNA preparation.

### 2.7. Exonuclease Resistance Assay 

RNA samples (500–1000 ng) were treated with enzymes in the experimental groups, while untreated RNA served as a control. The reaction mixture contained 2.5 U of RppH (5000 U/mL, New England Biolabs, Ipswich, MA, USA), 0.5 U of XRN1 (1000 U/mL, New England Biolabs, Ipswich, MA, USA), and 1 μL of 10× NEBuffer 3 (New England Biolabs, Ipswich, MA, USA). RppH was used to remove pyrophosphate from the 5′ end of triphosphorylated RNA, generating 5′-monophosphorylated RNA suitable as a substrate for XRN1 cleavage. Control reactions lacking the enzyme contained 1 µL of 10× NEBuffer 3 (New England Biolabs, Ipswich, MA, USA) and RNA, with the final volume adjusted using DEPC-treated water. The total reaction volume was 10 μL. The mixtures were incubated at 37 °C for 1 h. The enzymatic reaction was halted by the addition of 10 μL of 2× RNA Sample Buffer (New England Biolabs, Ipswich, MA, USA).

### 2.8. Denaturing Polyacrylamide Gel Electrophoresis (PAGE)

Electrophoresis was performed using a denaturing polyacrylamide gel consisting of 1× TBE buffer, 8% (19:1) acrylamide/bis-acrylamide, 6.5 M urea, 0.08% ammonium persulfate, and 0.04% TEMED. The gel was pre-run in a 1× TBE buffer for 1 h at 300 V. Subsequently, 500–1000 ng of each RNA sample and 10 μL of RNA marker—both preheated at 95 °C for 5 min—were loaded. The gel was stained with ethidium bromide in 1× TBE buffer for 10 min and visualized using a gel documentation system (Vilber, Paris, France).

## 3. Results

Previously, a method was proposed and validated for most orthoflaviviruses to study the resistance of 3 genomic RNAs to XRN1 in vitro [17]. This method involves treating the target RNA with XRN1 and RppH, followed by the separation of the experimental and control samples via polyacrylamide gel electrophoresis. It is expected that the protected XRN1-resistant RNA will exhibit a shorter length than the initial full-length RNA, indicating the presence of structures that prevent complete hydrolysis. Non-resistant RNA, however, will be fully degraded by XRN1 exoribonuclease.

**Figure 1:**
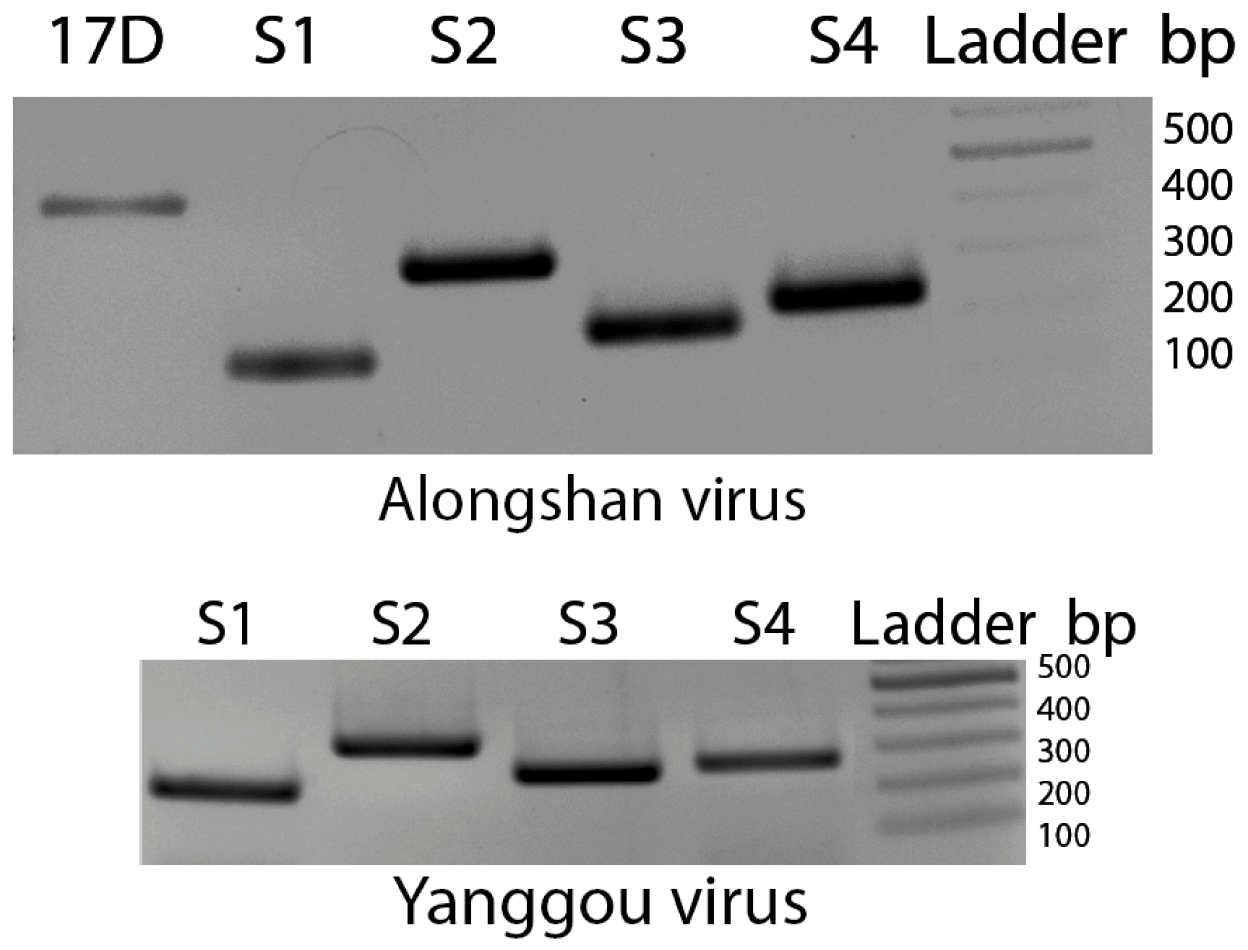
Electropherogram of target PCR amplicons of yellow fever virus and all genomic segments of Alongshan and Yanggou tick viruses. 17D—yellow fever virus 17D; S1–S4—Segments 1–4.

First, PCR products were amplified using the specified primers for subsequent cloning. The presence of the target inserts, comprising the 3′ UTR of yellow fever virus and all four segments of Alongshan and Yanggou tick viruses, was confirmed via analytical agarose gel electrophoresis (Figure 1).

The sizes of the target amplicons, which included the T7 phage promoter sequence, were consistent with the expected lengths (Table 2). Subsequently, Sanger sequencing of the recombinant plasmid clones confirmed the absence of unintended mutations (Figure S1).

**Figure 2:**
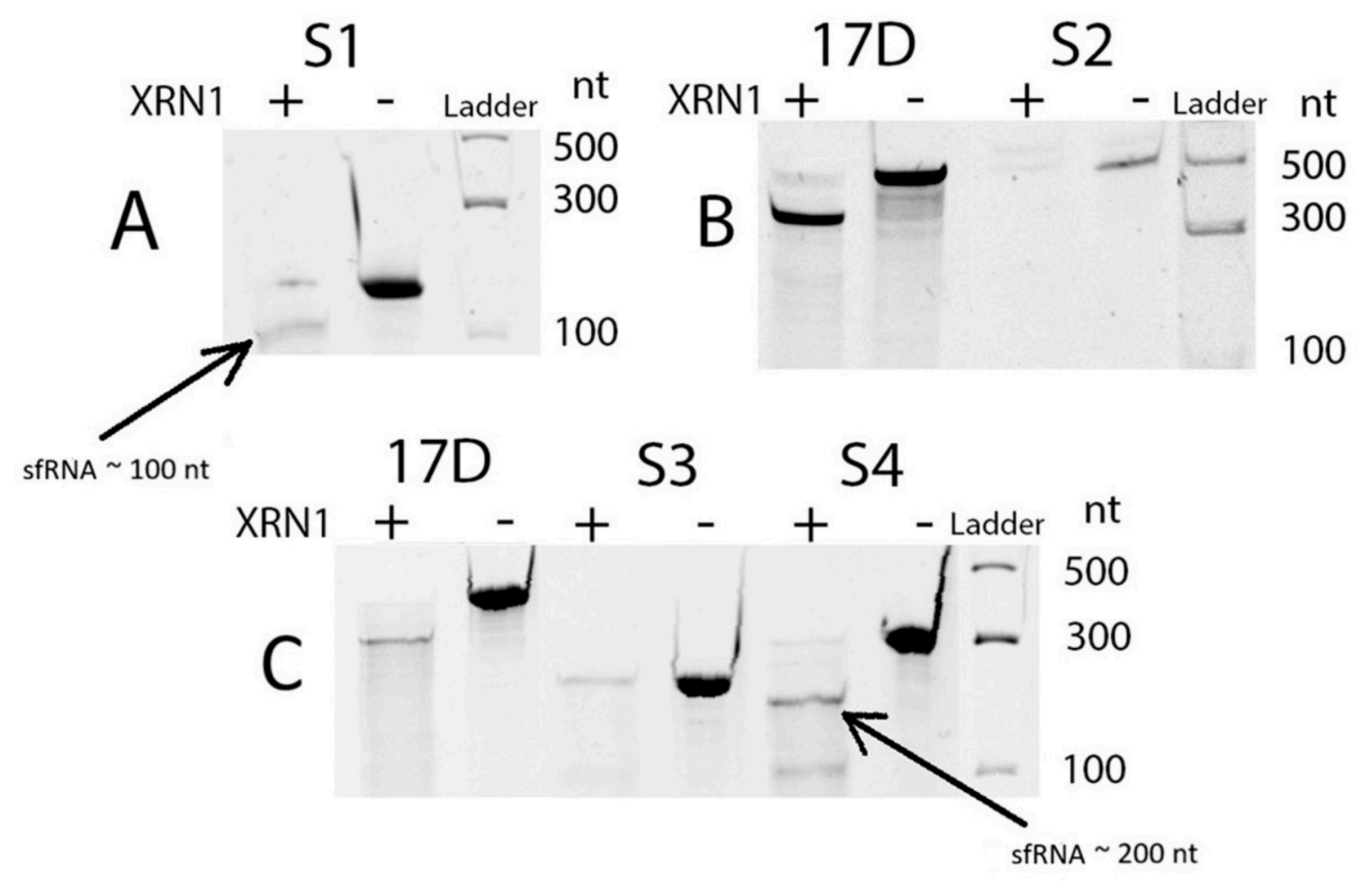
First independent replicate. 8% PAGE analysis of yellow fever virus 3′ UTR RNA and Alongshan virus genomic segments. «+» RppH/XRN1 treatment; «−» untreated control. (**A**) Segment 1; (**B**) yellow fever virus 17D, segment 2; (**C**) yellow fever virus 17D, segment 3 and segment 4.

To evaluate resistance to XRN1 exonuclease, RNA fragments corresponding to the 3′ untranslated regions were synthesized using linearized plasmid DNA as a template. The in vitro obtained 3′ UTR of the yellow fever virus, which is known to generate sfRNAs [7], served as a positive control. The incomplete digestion of the yellow fever virus 3′ UTR validated the functionality of the exonuclease resistance assay (Figure 2).

Following enzymatic analysis and separation by PAGE, it was determined that the in vitro 3′ UTR of RNA segments 1 and 4 of the Alongshan virus displayed incomplete hydrolysis compared to the untreated control; specifically, the band in the treated sample migrated lower than that of the untreated control (Figure 2A,C). In contrast, segments 2 and 3 showed complete RNA degradation, with no discernible band present in the experimental lane at or below the expected size RNA (Figure 2B,C).

These data indicate the presence of XRN1 exonuclease resistance in specific segments. The resistance of segments 1 and 4 to complete enzymatic digestion suggests the presence of stable secondary RNA structures at the 3′ end that impede XRN1 translocation. To ensure reproducibility, all experiments were performed in triplicate (Figure S2).

**Figure 3:**
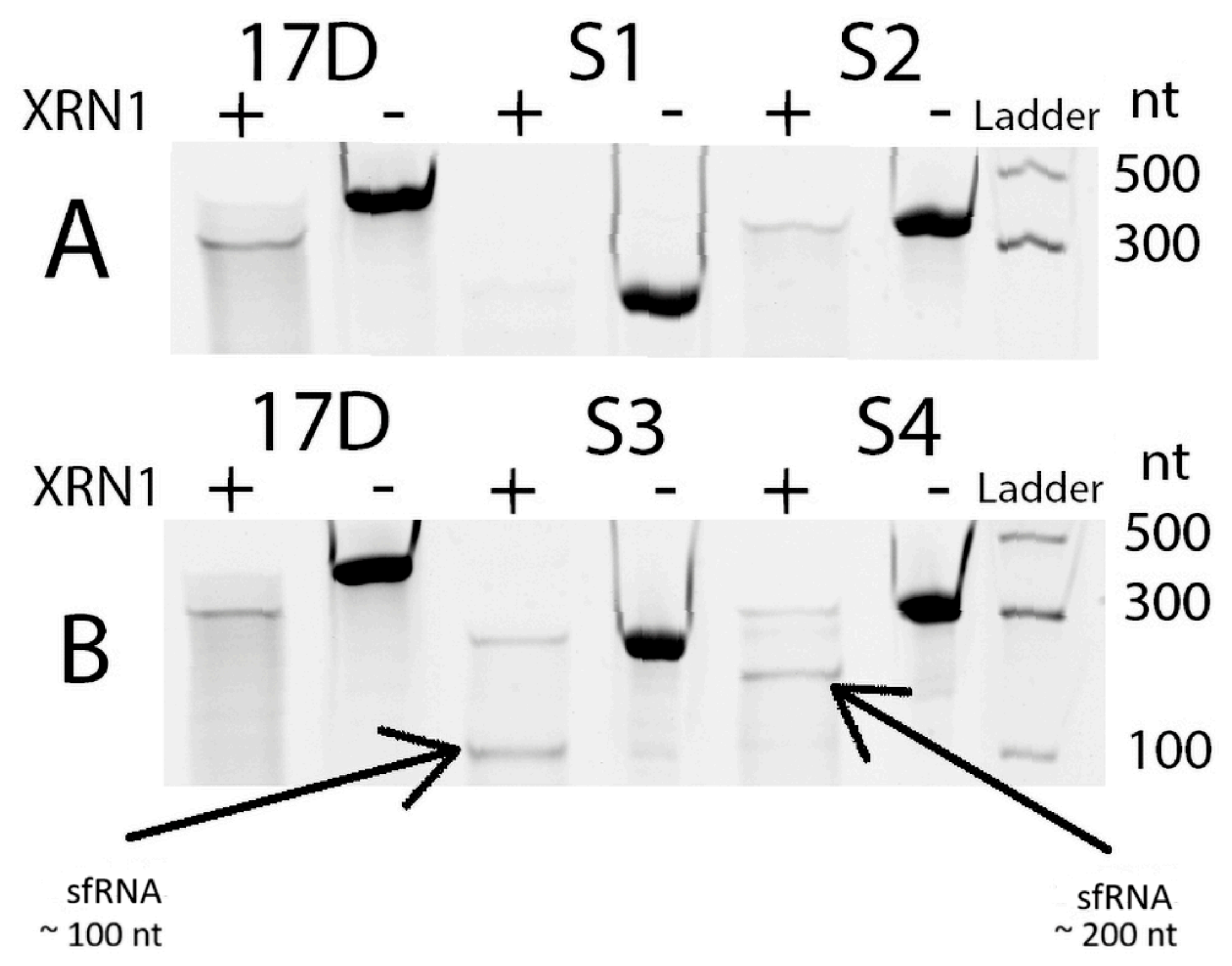
First independent replicate 8% PAGE analysis of yellow fever virus 3′ UTR RNA and Yanggou tick virus genomic segments. «+» RppH/XRN1 treatment; «−» untreated control. (**A**) yellow fever virus 17D, segment 1 and segment 2; (**B**) yellow fever virus 17D, segment 3 and segment 4.

An experiment with the Yanggou tick virus 3′ UTR RNA yielded similar results. However, in this case, resistance to XRN1 was demonstrated by segment 3 in addition to segment 4 (Figure 3). These experiments were also performed in triplicate and showed consistent results (Figure S3).

It is important to note that the sizes of the subgenomic RNAs were approximately comparable between Alongshan virus and Yanggou virus, between segments four and four and one and three, respectively.

## 4. Discussion

As previously established, the orthoflavivirus genome is flanked by a capped 5′ UTR, which mediates translation initiation, and a relatively long, highly structured, non-polyadenylated 3′ UTR. The RNA architecture of the distal portion of the 3′ UTR remains relatively conserved across the genus *Orthoflavivirus*. This region harbors conserved RNA sequences that facilitate base-pairing with complementary sequences at the 5′ terminus, a process critical for viral genome circularization and replication [17].

The 3′ UTR contains several stem-loop (SL) structures and pseudoknots that exhibit high conservation, particularly among orthoflaviviruses within the same subgroup. This domain is responsible for the accumulation of non-coding subgenomic flaviviral RNAs (sfRNAs) in infected cells. This accumulation results from the stalling of the cellular exoribonuclease XRN1 at specific exoribonuclease-resistant RNA (xrRNA) structures following the degradation of approximately 95% of the orthoflavivirus genome [7,8]. These sfRNAs are involved in the hydrolysis of cellular RNA, the regulation of RNA interference pathways, and the modulation of the host immune response [10,11]. Furthermore, XRN1 activity is diminished following orthoflavivirus infection, suggesting that the enzyme may be sequestered via stalling on xrRNA structures, thereby shifting cellular RNA homeostasis to favor viral replication [18].

XRN1-resistant elements are characterized by conserved secondary and tertiary structures within each orthoflavivirus clade [8,19,20]. To date, most characterized orthoflavivirus sfRNAs feature stem-loop structures containing a three-way junction (3WJ) formed by three RNA helices. Pseudoknots and non-canonical intra-sfRNA interactions define a unique tertiary conformation in which the 5′ terminus of the RNA passes through a ring-like structure. Specifically, nucleotides A37(10401) and U51(10415) within the 71-nt xrRNA1 of the Zika virus form a long-range reverse Watson–Crick (trans) base pair, which closes the ring and effectively “hooks” the internal RNA strand. [21]. This unique conformation confers XRN1 resistance, as the RNA ring creates a physical barrier to the translocation of the enzyme [21,22].

Secondary structures have been experimentally determined for mosquito-borne flaviviruses (MBFVs) [19] and tick-borne orthoflaviviruses (TBFVs) [20]. Additionally, crystal structures have been resolved for three MBFV sfRNAs [21] and the sfRNA of the bat-associated Tamana virus (TABV) [22]. Based on these conserved motifs, sfRNAs are categorized into two classes: Class 1, present in MBFVs, and Class 2, found in TBFVs and NKVs [8].

The key distinction lies in the length of Pseudoknot 1 (PK1): Class 1 xrRNAs contain a short PK1 consisting of 1–3 base pairs, whereas Class 2 xrRNAs possess an elongated PK1 of 6–10 base pairs [8,23]. Consequently, the XRN1 halt site occurs upstream of the ring-like structure in Class 1, but maps within the elongated PK1 element in Class 2, suggesting a distinct stalling mechanism [8].

The 3′ UTRs of MBFVs typically contain duplicated SL-based sfRNA structures, followed by two dumbbell motifs and a terminal 3′ stem-loop [23,24]. These duplicated SLs give rise to two sfRNA isoforms of varying lengths [24]. Furthermore, MBFVs produce two small sfRNAs, with their 5′ termini likely positioned at the start of the dumbbell structures. However, the biogenesis of these specific RNAs remains poorly understood, as recent evidence suggests that dumbbell structures lack intrinsic XRN1 resistance [25]. It remains unclear why MBFVs acquired additional sfRNAs or whether these isoforms possess specialized functions. Notably, dengue virus 2 (DENV-2) accumulates mutations in individual sfRNAs and switches from longer to shorter isoforms during host adaptation [26]. This suggests that the duplication of structural elements within the 3′ UTR represents an adaptation to the dual-host life cycle, with specific sfRNA species optimized for functioning within different host cell environments. However, this functional specialization has only been definitively shown for DENV-2, and the hypothesis remains to be fully validated across other species. To date, the in vivo generation of sfRNAs from the 3′ UTR has been demonstrated only for the genera *Orthoflavivirus* [27]. For other genera, sfRNA generation has been observed exclusively in vitro [27].

Jingmenviruses are of particular interest due to their unique genomic organization. The study of segmented (+) RNA genomes in viruses transmitted by arthropod vectors provides novel insights into virus–host factor interactions. Moreover, these viruses, particularly the Alongshan virus (ALSV), are of significant medical importance [15].

Previously, we found several highly conserved regions in the 3′ UTRs of Jingmen viruses, with RNA-folding algorithms suggesting that these regions are involved in the formation of RNA secondary structures [14]. Here, in vitro cleavage assays demonstrate XRN1 stalling within the 3′ UTR segments of ALSV (in segments 1 and 4) and YGTV (in segments 3 and 4). However, the present work does not define the exact nucleotide boundary of XRN1 resistance, nor does it experimentally confirm the predicted folds. We acknowledge that further experimental validation—including fine mapping of the sfRNA 5′ ends by primer extension, mutational analysis disrupting predicted base-pairing, and high-resolution chemical probing (e.g., SHAPE-MaP)—will be required to pinpoint the exact minimal xrRNA element and elucidate its precise structural architecture.

Our study demonstrates the probable in vitro generation of subgenomic RNAs by Jingmenviruses. To our knowledge, this is the first such report for the genus *Jingmenvirus*. These data warrant further comprehensive investigation into the molecular biology of Jingmenviruses, specifically regarding the generation and function of sfRNAs in both tick and mammalian cellular systems.

## Figures and Tables

**Table 1:** Primer sequences used in this study.

Primer	Sequence
BHT7_3UTR_seg1	5′-ATGTAATACGACTCACTATAGGCGGTATTCAACTAATCAAGACA-3′
BHT7_3UTR_seg2	5′-ATGTAATACGACTCACTATAGGCTACTCGCGACTAGATC-3′
BHT7_3UTR_seg3	5′-ATGTAATACGACTCACTATAGGGAGCCCACTTAGAGGTG-3′
BHT7_3UTR_seg4	5′-ATGTAATACGACTCACTATAGGGAGTAGAGTAACATAGATG-3′
Miass519_seg1_CR	5′TTTGACTTGCGCCGTG-3′
Miass519_seg2_CR	5′TTGACTCCGGTGGACC-3′
Miass519_seg3_CR Miass519_seg4_CR	5′-TTGCAACGGGCATAGT-3′ 5′-TGTTCATCTTCCGGAAC-3′
BHT7_YGTV_seq1	5′-ATGTAATACGACTCACTATAGGATGGCGGTATTTCACTAAG-3′
BHT7_YGTV_seq2	5′-ATGTAATACGACTCACTATAGGGTATCTCGCGCCTAG-3′
BHT7_YGTV_seq3	5′-ATGTAATACGACTCACTATAGGGAGCCCACTTAGAGGTG-3′
BHT7_YGTV_seq4	5′-ATGTAATACGACTCACTATAGGTGGAGTAGGGTCACATAG-3′
YGTV_seq1_CR	5′-TTYCTCCTTAGTTCCTCC-3′
YGTV_seq2_CR	5′-TTGTAGGCTTGTGTCGA-3′
YGTV_seq3_CR	5′-TGGTTCTTACCTGGCG-3′
YGTV_seq4_CR	5′-TGTGTATCTCCGACGAG-3′
YFV_3`NTO_F	5′-TAATACGACTCACTATAGGGAAACACCATCTAACAGG-3′
YFV_3`NTO_R	5′-AGTGGTTTTGTGTTTGTCA-3′

The underlined sequence denotes the T7 promoter.

**Table 2:** Expected length of amplicons.

Amplicon Name	Size (nt)
yellow fever virus 17D	529
Segment 1 of Alongshan virus	228
Segment 2 of Alongshan virus	396
Segment 3 of Alongshan virus	264
Segment 4 of Alongshan virus	300
Segment 1 of Yanggou tick virus	237
Segment 2 of Yanggou tick virus	359
Segment 3 of Yanggou tick virus	282
Segment 4 of Yanggou tick virus	304

## Data Availability

The original contributions presented in this study are included in the article/Supplementary Material. Further inquiries can be directed to the corresponding author.

## Supplementary Materials

The following supporting information can be downloaded at https://www.mdpi.com/article/10.3390/v18090946/s1, Figure S1: Cloning scheme; Figure S2: Replications of experiments to determine the stability of segments 1 and 4 of Alongshan virus; Figure S3: Replications of experiments to determine the stability of segments 3 and 4 of Yanggou tick virus.
