# Supplementary material for: XRN1-Resistant Elements Are Located in the 3′-Terminal Regions of Certain Genome Segments of Jingmenviruses
Source: Viruses. 2026 Aug 29;18(9):946. doi: 10.3390/v18090946 (PMC13612070; doi:10.3390/v18090946)

Figure S1. Cloning scheme.

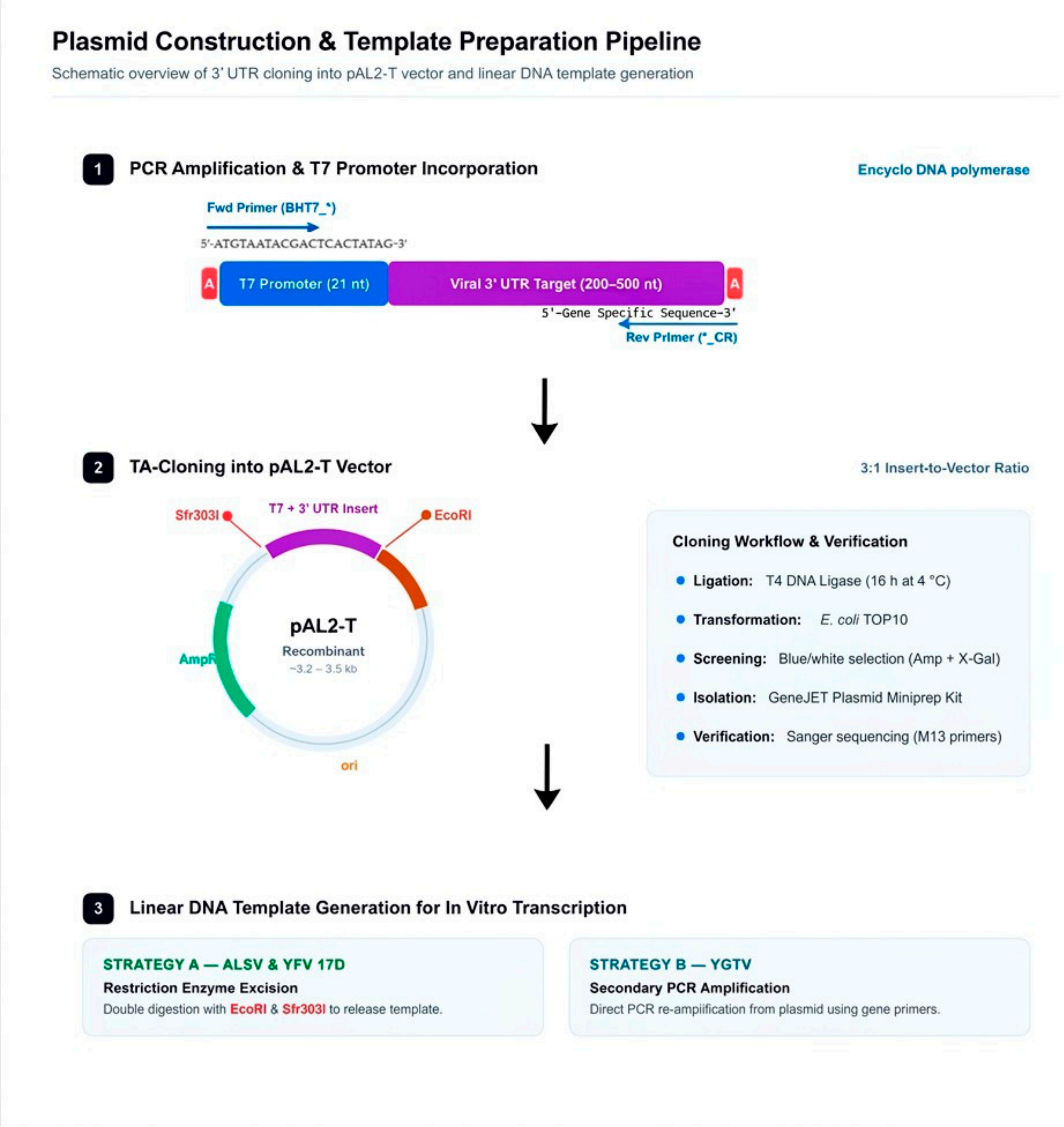

Supplement: Supplementary file 1 [file viruses-18-00946-s001.zip › Figure S1.pdf]
